# Synthesis and Characterization of Fatty Acid/Amino Acid Self-Assemblies

**DOI:** 10.3390/jfb5040211

**Published:** 2014-10-24

**Authors:** Joanna Gajowy, Durgadas Bolikal, Joachim Kohn, Miroslawa El Fray

**Affiliations:** 1 Department of Biomaterials and Microbiological Technologies, The West Pomeranian University of Technology, Szczecin, Al. Piastow 45, 70-311 Szczecin, Poland; E-Mail: jgajowy@zut.edu.pl; 2 New Jersey Center for Biomaterials, Rutgers, The State University of New Jersey, 145 Bevier Road, Piscataway, NJ 08854, USA; E-Mails: bolikal@dls.rutgers.edu (D.B.); kohn@rutgers.edu (J.K.)

**Keywords:** amphiphilic block copolymers, fatty acid, micelle

## Abstract

In this paper, we discuss the synthesis and self-assembling behavior of new copolymers derived from fatty acid/amino acid components, namely dimers of linoleic acid (DLA) and tyrosine derived diphenols containing alkyl ester pendent chains, designated as “R” (DTR). Specific pendent chains were ethyl (E) and hexyl (H). These poly(aliphatic/aromatic-ester-amide)s were further reacted with poly(ethylene glycol) (PEG) and poly(ethylene glycol methyl ether) of different molecular masses, thus resulting in ABA type (hydrophilic-hydrophobic-hydrophilic) triblock copolymers. We used Fourier transform infrared (FTIR) and nuclear magnetic resonance (NMR) spectroscopies to evaluate the chemical structure of the final materials. The molecular masses were estimated by gel permeation chromatography (GPC) measurements. The self-organization of these new polymeric systems into micellar/nanospheric structures in aqueous environment was evaluated using ultraviolet/visible (UV-VIS) spectroscopy, dynamic light scattering (DLS) and transmission electron microscopy (TEM). The polymers were found to spontaneously self-assemble into nanoparticles with sizes in the range 196–239 nm and critical micelle concentration (CMC) of 0.125–0.250 mg/mL. The results are quite promising and these materials are capable of self-organizing into well-defined micelles/nanospheres encapsulating bioactive molecules, e.g., vitamins or antibacterial peptides for antibacterial coatings on medical devices.

## 1. Introduction

Synthetic polymers have played an important role in medical therapies, being applied in areas such as modulation of wound healing, implantable medical devices and artificial organs, prostheses, ophthalmology, dentistry, bone repair, and drug delivery systems. Polymeric biomaterials are relatively easy to manufacture into products with various shapes, at reasonable cost, and with desirable mechanical and physical properties. However, one of the major factors limiting the use of these materials is their biocompatibility. In particular, the design of biocompatible synthetic surfaces that are able to control the interactions between living systems and the implanted material remains the main theme of applications of biomaterials in medicine [1].

The most common complications associated with polymeric implants are microbial infections. The most common hospital infections occur at four major body sites, leading to their description by the US Centers for Disease Control and Prevention (CDC) as the “Big Four”, namely surgical site infections (SSIs), pneumonia (PNEU), bloodstream infections (BSIs) and urinary tract infections (UTIs) [2].

Considering biomaterial-related infections, the best method of their protection against bacteria is to apply surface modification. It plays an important role in medical applications, because it provides specific biofunctionality (for example, lubricity) with antimicrobial activity [3]. There are various approaches being applied in surface modification of biomaterials, including chemical modification [4,5], surface grafting [3,4,6,7], self-assembled monolayers [4,8,9], plasma treatments [4,7,10], E-beam and gamma radiation [4,7,10], ultraviolet (UV) light [4,10], corona and laser treatments [7], and blending [7].

Chemical or physical modification of biomaterials surfaces is the most common method. Synthetic and natural polymers are being used:* i.e.*, poly(ethylene glycol) (PEG)-based polymers [9,11], poly-(2-hydroxyethyl methacrylate)(PHEMA) [5], poly(2-methacryloyloxy-ethylphosphorylcholine-*co*-*n*-butyl methacrylate)(poly(MPC-*co*-BMA)) [12], amphiphilic phospholipid polymers [9], poly(2-methacryloyloxyethyl phosphorylcholine) brush [13] or chitosan [14]. An interesting group of natural-origin materials are long-chain unsaturated fatty acids, which have been well known as growth inhibitors for microorganisms on the surface of medical devices [15]. The long-chain, unsaturated fatty acids, including oleic, linoleic, and linolenic acid, exhibit better bactericidal properties than do long-chain, saturated fatty acids, including palmitic and stearic acid [15]. Whether saturated or not, medium and long-chain fatty acids exhibit a broad spectrum of microbial activity against enveloped viruses and various bacteria and fungi* in vitro*, including various pathogens, herpes simplex virus (HSV) [16], *Neisseria gonorrhoeae* [16], group B *streptococci* (GBS), group A *streptococci* (GAS), *Staphylococcus aureus* [15,16,17], to mention a few. Amino acids and their derivatives, which are the basic components of polypeptides, play an important role in the human body. Amino acid-based polymers exhibit a wide potential for applications as biocompatible materials. Some of these polymers have unique properties and functions derived from amino acid moieties alone [18]. For example, molecules containing phenolic hydroxyl functions and amino acids such as cysteine, methionine and tyrosine are known to exert an antioxidant effect in humans [19]. It was shown that they have a significant effect in: increased antibacterial activity of the VO(IV) mixed-ligand complexes of ciprofloxacin and dl-alanine (or l-tyrosine or l-tryptophan or glutamic acid or l-leucine) against *Staphylococcus aureus*, *Baccilus subtilis*, *Serratia marcescens* (Gram-positive), and *Pseudomonas aeruginosa*, and *Escherichia coli* (Gram-negative) bacteria [20]. In our work, we focus on l-tyrosine, which is the only major, natural nutrient containing an aromatic hydroxyl group. The amino-acids, in this case l-tyrosine, can be linked by non-amide bonds, such as ester, iminocarbonate and carbonate bonds to form tyrosine-derived pseudopoly(amino acid)s [21]. Furthermore, polymers containing pseudo-poly(amino acid)s can exhibit high stability, nanometer sized structure and self-organized molecular character, which is very attractive for medical applications [21,22,23,24,25].

Therefore, the combination of appropriate fatty acid (here dimerized fatty acid, DLA [26,27,28,29] and tyrosine (Tyr) derivative and PEG should provide new materials with specific desired properties.

**Figure 1:**
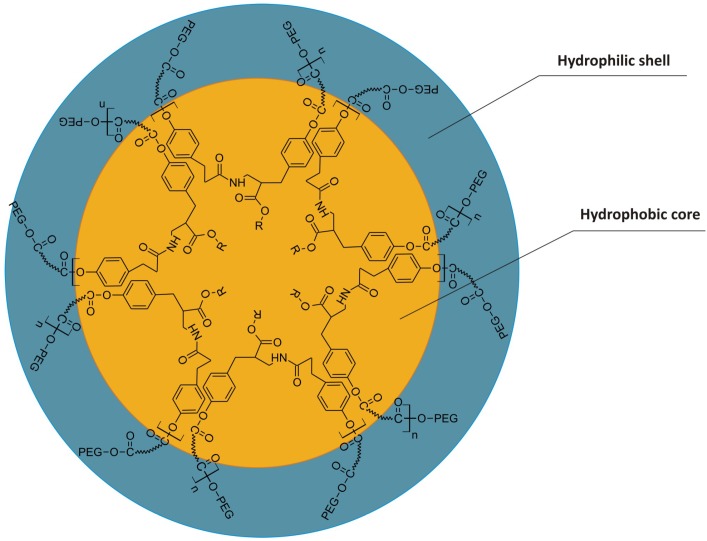
Model ofmicelle capableto encapsulatevitamins/antimicrobial peptides in a core.

The major goal of this work is to evaluate the chemical structure of new copolymers and their self-organization into micelles/nanospheres. These structural components were coupled with poly(ethylene glycol) (PEG) of different molecular masses, thus providing poly(aliphatic/aromatic-ester-amide-ether)s. Their amphiphilic character was determined by hydrophilic shell composed of PEG and the hydrophobic core of dimerized fatty acid (DLA)/Tyr. The schematic cross-section of DLA/Tyr-PEG micelle, capable of self-assembling and potentially encapsulating bioactive components such as vitamins or antimicrobial peptides is shown in Figure 1.

## 2. Experimental Section

### 2.1. Materials

**Figure 2:**
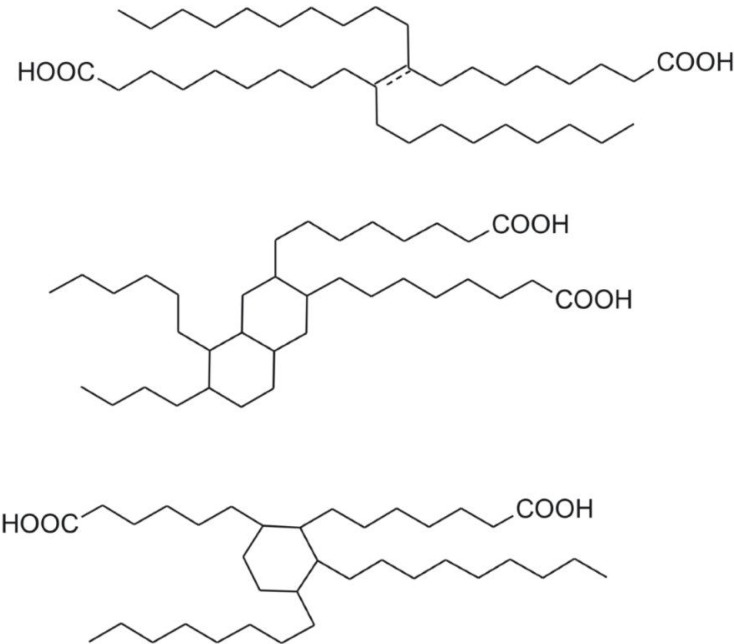
Structures of dimerized fatty (linoleic) acid.

Tyrosine-derived monomers (DTR) were kindly provided by the New Jersey Center for Biomaterials at Rutgers University (Piscataway, NJ, USA). Dimerized fatty acid, a hydrogenated DLA, trade name Pripol 1009 [30,31], of molecular weight ~570 g·mol^−1^, (970 g·mol^−1 ^from GPC, with dispersity index of 1.04), –COOH number 196 mg/g, was kindly provided by Croda, (Cowick Hall Snaith, East Yorkshire, UK). Some possible structures of Pripol are shown in Figure 2. Selected properties of this material are summarized in Table 1. 1-Ethyl-3-(3-dimethyllaminopropyl) carbodiimide hydrochloride (EDC·HCl) was obtained from GenScript (GenScript USA Inc., Piscataway, NJ, USA) Biology CRO for Drug Discovery. PEG and poly(ethylene glycol) methyl ether (mPEG) of different molecular masses were obtained from Aldrich Chemical (Sigma-Aldrich Chemie GmbH, Buchs SG, Switzerland) and dried before using according with procedure described in [32], Other chemicals such as polyvinyl alcohol 30–70 kDa, and 4-dimethylaminopyridine (DMAP), were obtained from Aldrich Chemical. All solvents of high performance liquid chromatography (HPLC) grade were used without further purification.

**Table 1:** Molecular weight and dispersity index of the PAAEAEs.

Polymer	*M*_n_(Da)	*M*_w_ (Da)	Dispersity index
DTE_DLA_mPEG5000	38,700	83,300	2.15
DTE_DLA_PEG6000	35,400	66,300	1.87
DTH_DLA_mPEG5000	21,400	48,800	2.28
DTH_DLA_PEG6000	17,100	40,000	2.32

### 2.2. Synthesis

**Figure 3:**
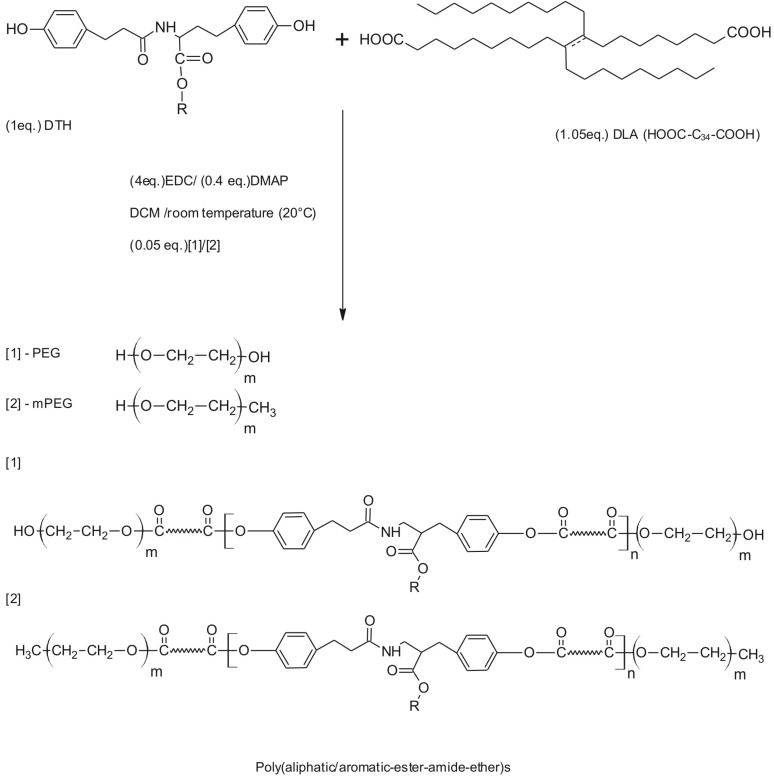
Synthesis scheme of poly (aliphatic/aromatic-ester-amide-ether)s.

Poly(aliphatic/aromatic-ester-amide-ether)s (PAAEAE) were synthesized using 1-ethyl-3-(3-dimethyllaminopropyl) carbodiimide hydrochloride (EDC·HCl) as the coupling agent and 4-dimethylaminopyridine (DMAP) as the catalyst, in dichloromethane solution (DCM), at room temperature, in an inert gas atmosphere. Briefly, poly(aliphatic/aromatic-ester-amide-ether)s were synthesized as follows: DLA (1.05 equivalent), DTE (desaminotyrosyltyrosine ethyl ester) or DTH (desaminotyrosyltyrosine hexyl ester)(1 equivalent) and DMAP (0.4 equivalent) were placed in a 100 mL round-bottomed flask. Then, dichloromethane was added and after compounds dissolved, 4 equivalent of EDC·HCl was added. The reaction mixture was cooled down to 0 °C and stirred for 2 h. The reaction mixture was removed from the ice bath and allowed to warm up to room temperature with continuous stirring at room temperature for 24 h. After 24 h, PEG or mPEG of molecular masses 1000 g/mol or 5000 g/mol was added to the reaction mixture and was continuously stirred for the next 24 h. Following 24 h of additional stirring, the reaction mixture was precipitated with 2-propanol. The precipitate was dried, dissolved in 10 mL of methylene chloride, and re-precipitated with 50 mL of methanol. This stage was also important to remove the initiator. The product was isolated by centrifugation and dried under vacuum at room temperature. The synthesis scheme of poly (aliphatic/aromatic-ester-amide-ether) (PAAEAE) copolymers is shown in Figure 3. The resulting copolymers were characterized by FTIR, ^1^H NMR (CDCl_3_, 400 MHz) and GPC.

### 2.3. Preparation of Nanospheres

Nanospheres were obtained as a result of self-assembling of polymer solution (20 mg of copolymer in 1–2 mL of tetrahydrofuran (THF) in 0.1% aqueous solution of polyvinyl alcohol PVA. The resulting turbid dispersion was sequentially filtered through 0.45 μm and 0.22 μm size syringe filters (PVDF, Millipore, Bedford, MA, USA) to remove particles greater than 220 nm in diameter. Trace organic solvent was removed by gentle nitrogen blow-drying. Polymeric self-assemblies were characterized by UV-Vis spectroscopy. Particle size and polydispersity index (PDI) of nanospheres were determined using dynamic light scattering (DLS).

### 2.4. Characterization Methods

#### 2.4.1. FTIR/^1^H NMR

The chemical structure of polymers was characterized by Fourier transform infrared spectroscopy (FTIR) and by nuclear magnetic resonance spectroscopy (^1^H NMR). FTIR spectra were obtained using the Thermo Nicolet NEXUS spectrometer (Thermo Fisher Scientific, Inc., Waltham, MA, USA), scanning between 500 and 4000 cm^−1 ^(resolution 4 cm^−1^, 32 scans). The polymeric material was spread onto a sodium chloride plate and transmission spectra were collected. EZ OMNIC software (Thermo Fisher Scientific Inc., Waltham, MA, USA), was used for analyzing data. ^1^H NMR spectra were obtained using the Varian VNMRS 400 MHz (Varian Medical Systems Inc., Palo Alto, CA, USA). CDCl_3_ was used as solvent and all shifts were determined with reference totetramethylsilane TMS. MestReNova software [33] was used for data analyzing.

#### 2.4.2. Gel Permeation Chromatography (GPC)

GPC experiments were performed to determine the molecular mass distribution and dispersity index. The GPC system consisted of a 515 HPLC pump, a 717 plus auto sampler, and a 410 RI detector (Waters Associates, Milford, MA, USA). Two PL gel columns 1 × 10^3 ^and 1 × 10^5^ Å (Polymer Laboratories, Santa Clara, CA, USA) were used in series with THF as the mobile phase at a flow rate of 1 mL/min. The samples were dissolved in THF to give a concentration of 10 mg/mL and 20 µL was injected. Waters Associates Empower 2 software [34] was used for data collection and molecular mass calculations. The molecular masses were computed against polystyrene standards of MW 523,000, 204,000, 96,000, 20,235, and 7200.

#### 2.4.3. UV-Vis for CMC Estimation

Micelles/nanospheres were characterized by UV-Vis spectroscopy. The absorbencies of the test solutions were recorded on UV-Visible Spectrophotometer Thermo Scientific (Thermo Fisher Scientific Inc., Waltham, MA, USA). All wavelength scans were performed at room temperature. The critical micelle concentration (CMC) of PAAEAEs copolymers in aqueous solution was determined using the hydrophobic dye solubilization method with 1,6-diphenyl-1,3,5-hexatriene (DPH) as indicator. DPH in concentration of 0.4 mM in methanol was used in CMC determination. Stock solutions of copolymers were prepared at a concentration of 1 wt%. Polymer solutions in the concentration range of 1.0% to 1.0 × 10^−5^% (w/V) were prepared by serial dilutions of the stock solutions. Then, 25 μL of the DPH solution was added to 2.5 mL of each polymer solution so that the final concentration of DPH was at 0.004 mM in each solution. The polymer solutions were incubated for 24 h in a dark place. The UV absorption of the solutions was measured at 356 nm and the absorbance values were plotted against copolymer concentration.

#### 2.4.4. Dynamic Light Scattering (DLS)

Particle size and polydispersity index (PDI) of obtained nanospheres were determined using dynamic light scattering DelsaNano S (Beckman Coulter Inc., Brea, CA, USA). Samples were measured at 25 °C at concentrations of approximately 1 mg/mL polymer. The suspensions were examined by normalized intensity distribution by the CONTIN method for cumulants, size distribution, and polydispersity. All measurements were performed in triplicate.

#### 2.4.5. TEM

The morphology of nanospheres was determined using transmission electron microscopy (TEM). For experiments with the negative staining of particles on grids (for virus, bacteria, small particles,* etc.*), a drop of the nanosphere dispersion was allowed to settle on a 200–400 mesh carbon/formvar coated grid and allowed to absorb to the formvar for a minimum of 1 min. The excess liquid was removed by gentle blotting with filter paper and a drop of staining solution (1% aqueous uranylacetate, Nanoprobes, Inc., Yaphank, NY, USA) was allowed to contact the sample for 1 min. The excess liquid was then wicked off and the grids allowed to dry. After the specimen was placed into the TEM, it was allowed to sit for a few minutes so that the sample could be vacuum dried before being irradiated. Electron micrographs were taken on a model JEM 100CX Transmission Electron Microscope (JEOL Ltd., Peabody, MA, USA).

## 3. Results and Discussion

### 3.1. Infrared Spectroscopy

**Figure 4:**
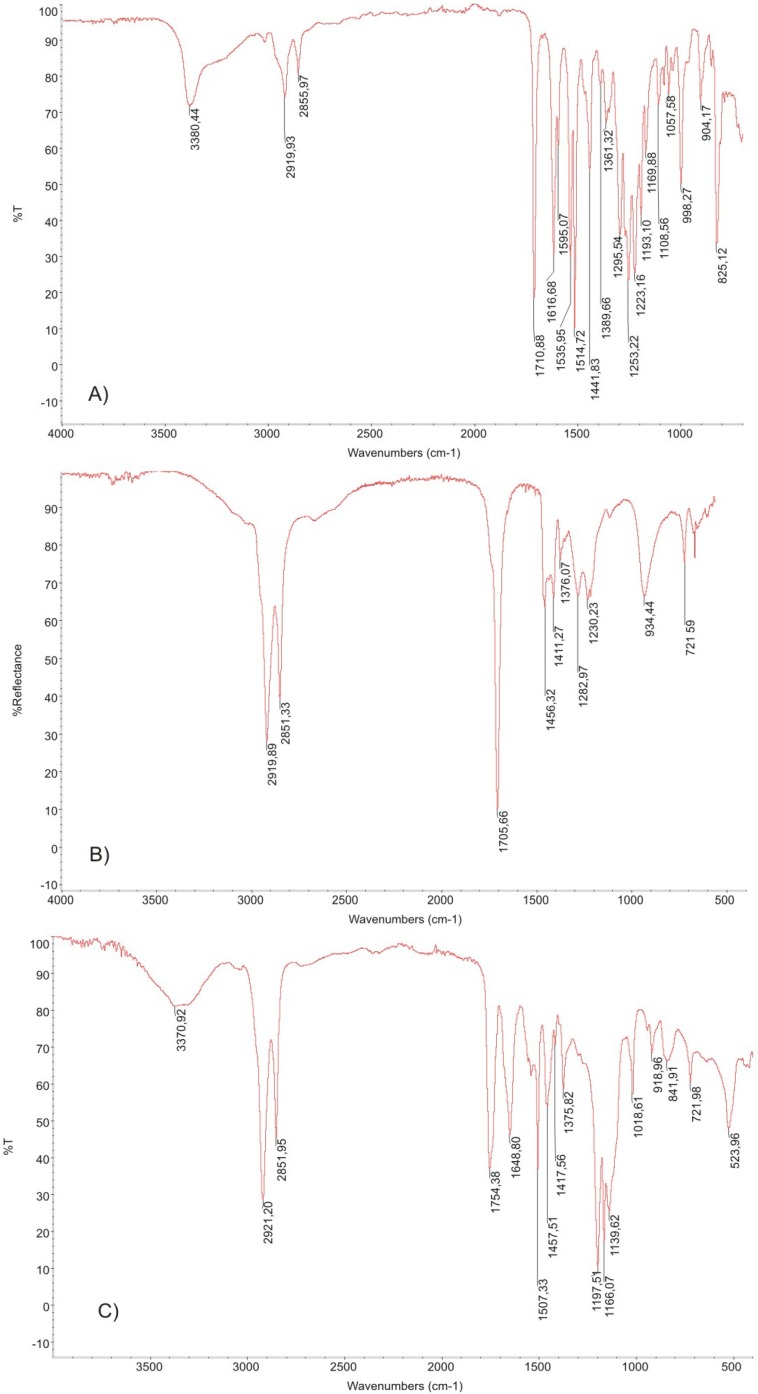
The FTIR spectra of initial reactants (**a**) DTE, (**b**) DLA and esterification product (**c**) DTE-DLA.

The chemical structure of new PAAEAE copolymers was verified with infrared spectroscopy. First, esterification of DTH (DTE) with DLA was performed and the functional groups were identified with FTIR spectroscopy (Figure 4). Based on FTIR spectrum of DTE derivative (Figure 4a) showed as an example, broad peak characteristic for Ar-OH group at 3380 cm^−1^ and intense peak characteristic for stretching vibrations of C=O group (1710 cm^−1^) can be seen, and they disappear after the reaction with DLA (its spectrum is presented in Figure 4c). An intense double-peak in the range 3000–2800 cm^−1^ is observed for DLA and it is ascribed to methylene –CH_2_– group. There is also an intense peak characteristic for stretching vibrations of C=O group (1705 cm^−1^). The spectrum for esterification product of DTR-DLA shows a new peak at 1754 cm^−1^ characteristic for stretching vibrations of the ester group. The disappearance of peaks at 1705–1710 cm^−1^ characteristic for Ar-OH and C=O groups is also observed.

**Figure 5:**
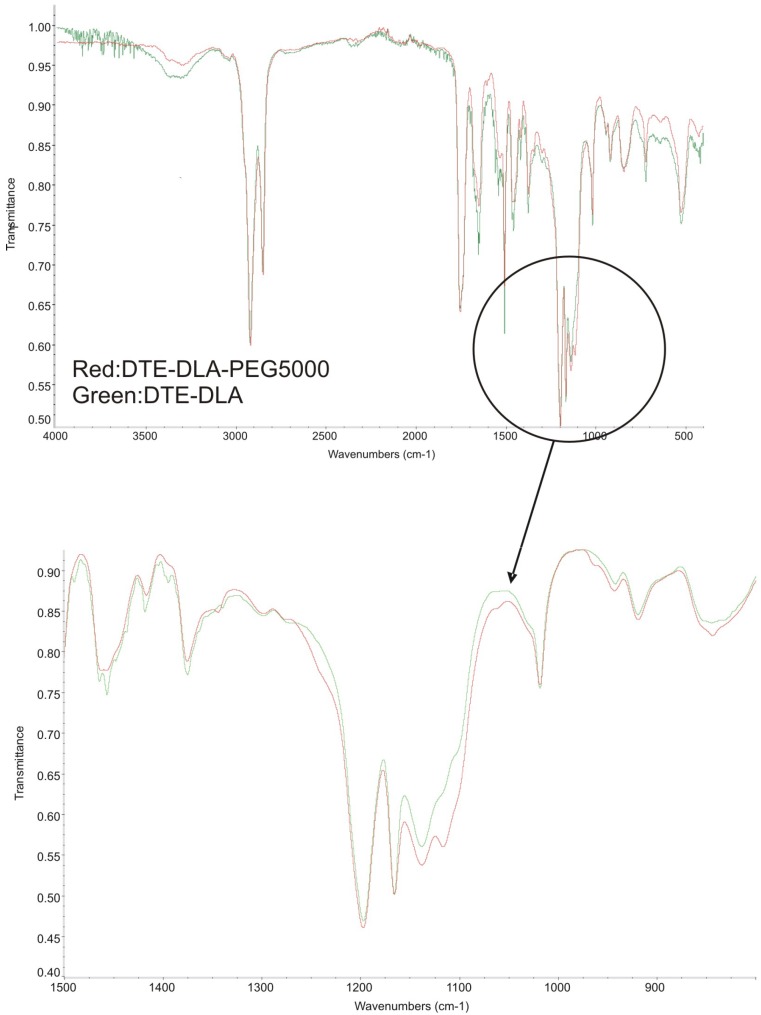
The representative spectrum of DTE_DLA_PEG5000.The expected polyethers, amide, ester, intense double-peaks characteristic for long methylene sequence in dimerized fatty acid are at 1112 and 1161, 1653, 1758, 2924 and 2853 cm^−1^, respectively.

The esterification product was further reacted with PEG or mPEG, and a representative spectrum for the final product, DTE_DLA_PEG5000 is shown in Figure 5. The analysis of FTIR spectra revealed occurrence of functional groups characteristic for esters, ethers and amides. The completion of the reaction and the final chemical structure was evidenced by the change in the intensity of bands characteristic for stretching vibrations of the ester and amide group, the presence of intense double-peaks (in the range 3000–2800 cm^−1^) which are characteristic for long methylene sequences in dimerized fatty acid, the presence of broad bands in the range of 3500–3000 cm^−1^ characteristic for –NH– group as well as bands in range of 1050–1200 cm^−1^ characteristic for stretching vibrations of the C–O groups derived from polyether. Higher magnification of 900–1500 cm^−1^ region shows the range characteristic for polyethers. As can be seen, an increased peak intensity at 1138 cm^−1^ as well as the appearance of a new, weak peak in the range of 1116 cm^−1^ characteristic for asymmetric stretching vibration of C–O–C group in aliphatic ethers was observed. These results presented in Figure 5 are in good agreement with FTIR spectra of pseudo-poly(amino acid)s containing poly(DTE-adipate) [35], where less intense double-peaks at 2940 cm^−1^ characteristic for shorter methylene groups related to adipic acid were found.

### 3.2. ^1^H NMR

**Figure 6:**
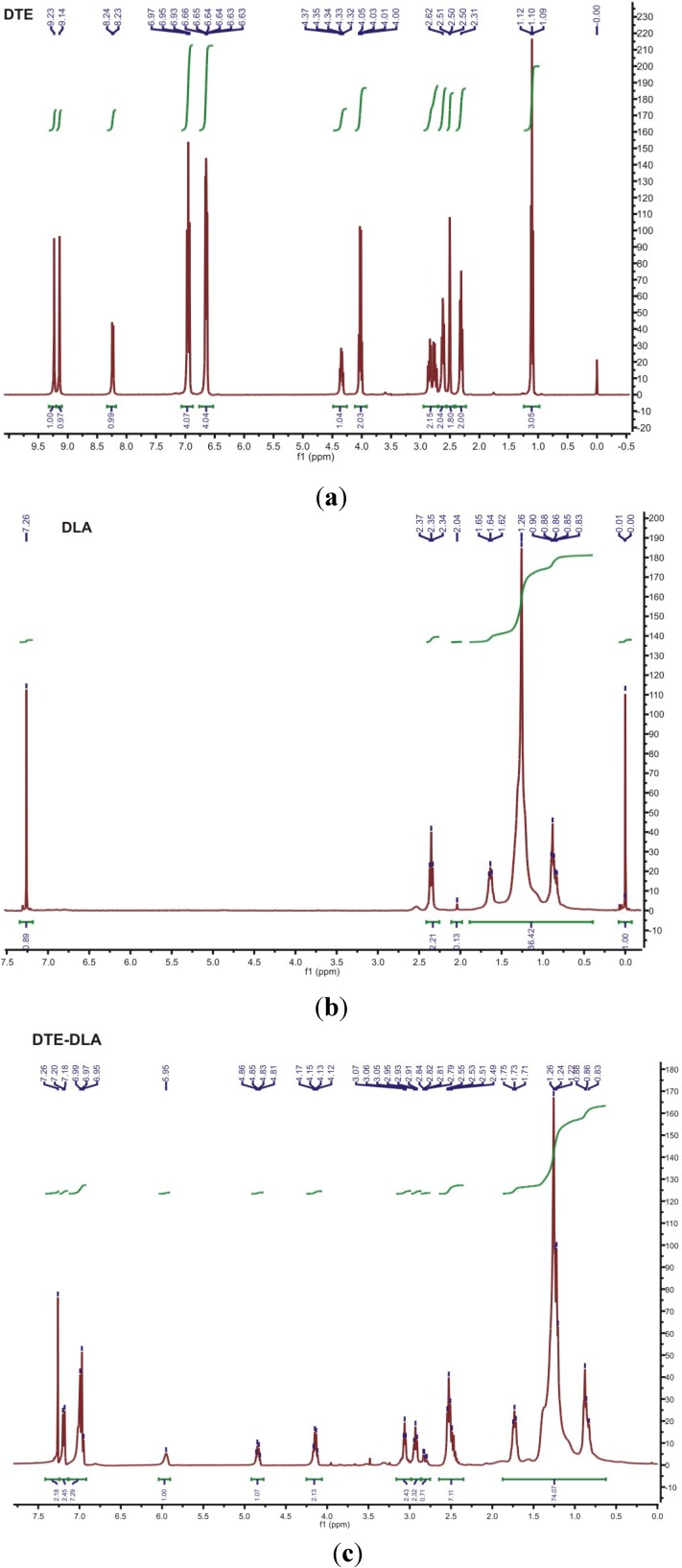
Spectra of initial reactants (**a**) DTE, (**b**) DLA and esterification product (**c**) DTE-DLA.

**Figure 7:**
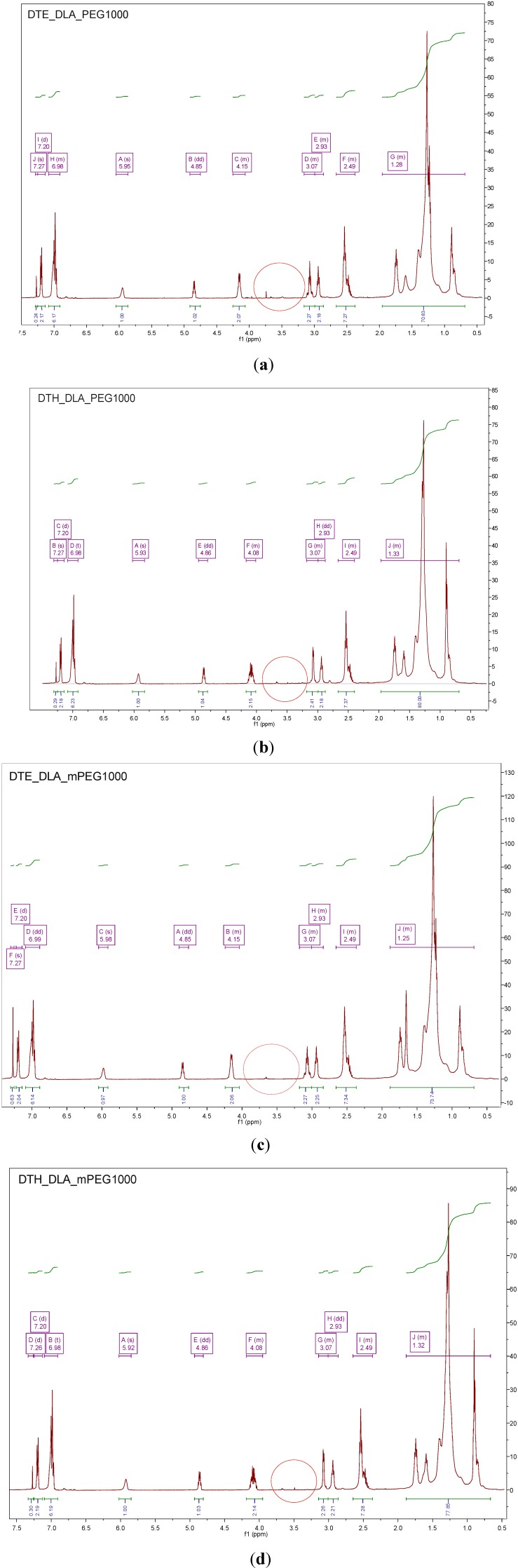
The ^1^H NMR spectra for poly(aliphatic/aromatic-ester-amide-ether)s (PAAEAE) containing (**a**) DTE_DLA_PEG 1000, (**b**) DTH_DLA_PEG1000, (**c**) DTE_DLA_mPEG 1000, and (**d**) DTH_DLA_mPEG1000 indicate absence of peaks in the range of 3.60–3.70 characteristic for these molecules.

The chemical structure of new copolymers was also characterized with ^1^H NMR. The detailed analysis of ^1^H NMR spectra for initial materials and esterification product is shown in Figure 6. The following shifts were found for DTE derivative: 9.23, 9.14 ppm (s, Ar-OH), 8.24–8.23 (d, –NH–). 6.97–6.63 ppm (Ar-H), 4.35 (dd, CH of tyrosine), 3.98 (t, –OCH_2_– of the pendent ester group), 2.81 ppm (ddd, –CH_2_–), 2.62 ppm (t, –CH_2_–), 2.51 (DMSO-d), 2.31 (t, –CH_2_–), 1.47, (m, –CH_2_–), 1.27 (m, –CH_2_–), 0.86 (t, –CH_3_). The detailed analysis of ^1^H NMR spectrum for DLA revealed the following shifts: 7.26 ppm (s, chloroform–d), 2.35 (t, –CH_2_–), 1.64 (t, –CH_2_–), 1.26 (m, –CH_2_–), 0.86 (m, –CH_3_). Finally, the DTE-DLA spectrum indicates the presence of a peak at 2.82 ppm characteristic for carbodiimide activation of carboxylic group in fatty acid. This peak disappears after reaction with PEG (Figure 7) along with simultaneous appearance of a characteristic signal at 3.65 ppm.

**Figure 8:**
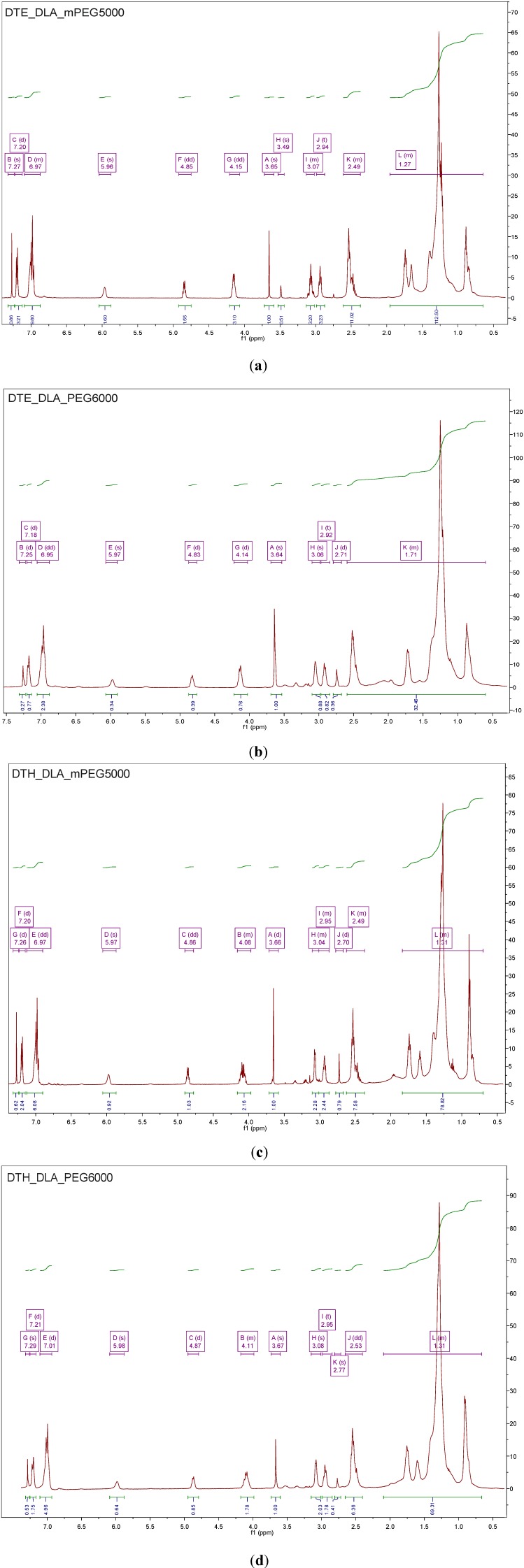
The ^1^H NMR spectra for PAAEAE containing (**a**) DTE_DLA_mPEG5000, (**b**) DTE_DLA_mPEG6000, (**c**) DTH_DLA_mPEG5000, and (**d**) DTH_DLA_PEG6000.

The analysis of ^1^H NMR PAAEAE containing PEG and mPEG of low molecular mass (1000 g/mol) (Figure 7) indicate the presence of peaks of low intensity (as marked by a circle), what is directly associated with low *M*n of this monomer. Therefore, more significant differences in chemical structure were observed when PEG and mPEG of higher molecular mass,* i.e.*, 5000 g/mol was used. The NMR spectra for polymeric materials containing PEG of low and high molecular masses are shown in Figure 7 and Figure 8, respectively. For DTE_DLA_mPEG5000, a short analysis of each peak was done.

The detailed analysis of ^1^H NMR spectrum for DTE_DLA_mPEG5000 material revealed the following shifts: 7.27 ppm (s, chloroform-d), 6.97–7.20 ppm (Ar-H), 5.96 (s, NH), 4.85 (dd, CH of tyrosine), 4.15 (dd, OCH_2_ of the pendent ester group), 3.65 (CH_2_CH_2_ of PEG), 3.49 (s, methanol), 3.07 (d, CH_2_ of DTE), 2.94 (t, CH_2_ of DTE), 2.49 (m, CH_2_ of DLA), 1.27 (m, CH_2_ of DLA). These results are in good agreement with NMR spectra of PEG-*b*-oligo(DTE-SA)-*b*-PEG triblock copolymer (where SA states for suberic acid and PEG of *M*_n_= 5000 g/mol was also used) [23].

### 3.3. GPC

**Figure 9:**
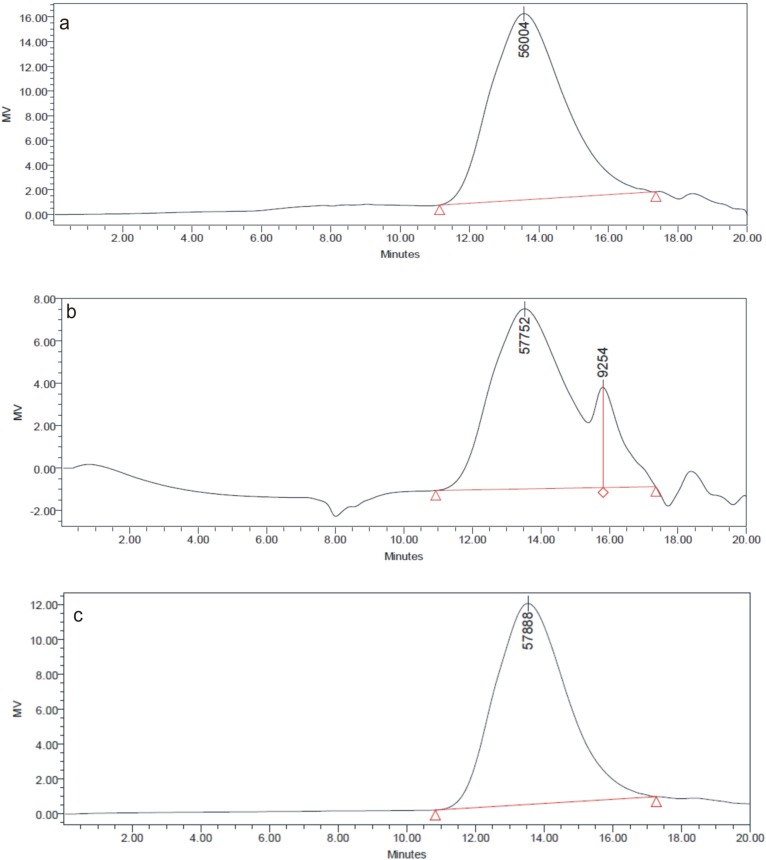
GPC chromatograms of esterification product (**a**) DTE-DLA, and the PAAEAE: after introducing PEG6000 (**b**) and for final, dry product DTE_DLA_PEG6000 (**c**).

The molecular weight of the final PAAEAEs copolymers made in the two-step processes are summarized in Table 1 and representative chromatograms for the esterification product (DTE-DLA) and the intermediate and final reaction products (DTE_DLA_PEG6000) are shown in Figure 9.

The *M*_n_ values for the PAAEAEs containing DTE (35 and 40 kDa) are higher compared to the DTH containing ones (21 and 17 kDa). The polydispersity index was around 2, which reflected the random copolymer structure. These differences in molecular masses of final materials are related to the complexity of the structure of the derivatives of tyrosine used in the reactions. Briefly, the more branched the structure of DTR molecules and the higher the molecular weight of PEG are, the lower the molecular weight of copolymers. Similar behavior was observed for triblock copolymers containing mPEG5000, DTR (DTE and DTO—octyl ester derivative of tyrosine) and SA [36] which showed lower or similar molecular weight in comparison to PAAEAE containing long-chain dimerized fatty acid.

### 3.4. UV-Vis

**Figure 10:**
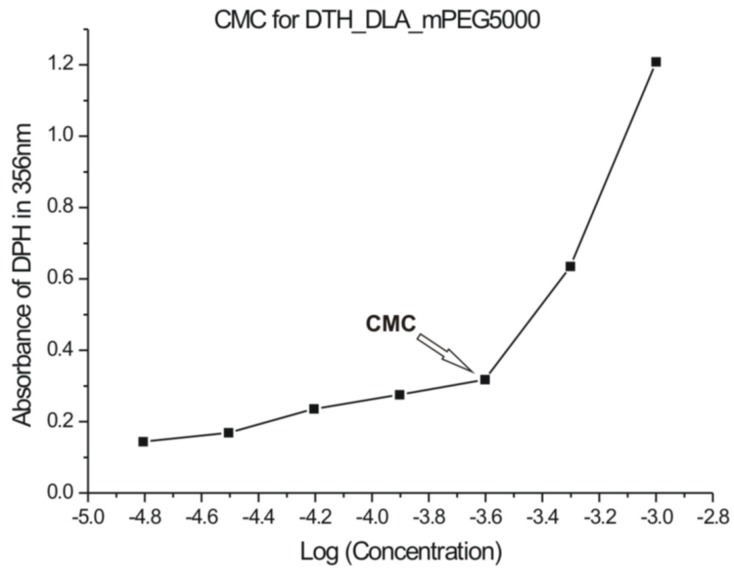
Absorbance of DPH at 356 nm in aqueous solution as a function of the DTH_DLA_mPEG5000 concentration selected at room temperature. The estimate of the critical micellization concentration from the data is indicated and the values are listed in Table 2.

The self-aggregation of amphiphilic PAAEAE in aqueous solution was characterized by UV-Vis spectroscopy. CMC of the copolymers were determined by a hydrophobic dye method, which has been employed for the characterization of amphiphilic block copolymers. The spectroscopic indicator 1,6-diphenyl-1,3,5-hexatriene (DPH) is a hydrophobic dye, which has a tendency to partition into the hydrophobic core of the micelles in the presence of amphiphilic block copolymers. It is known that the absorptivity of DPH at 356 nm in a hydrophobic environment is higher than in water [37]. The plot of absorbance* versus* copolymer concentration is shown in Figure 10. At low concentrations of copolymer in aqueous environment, the absorbance of DPH increases slowly. As the concentration of copolymer increased, the absorbance started to increase dramatically at a certain copolymer concentration, because the DPH completely dissolved in the hydrophobic core of copolymers corresponding to the micelles/nanospheres formation. CMC measurements were carried out at room temperature and the values are summarized in Table 2. The CMC values are higher for DTH containing PAAEAEs even though their molecular weights are lower, indicating that the micelle/nanosphere formation became easier for the copolymers with smaller hydrophobic blocks.

The standard free energy change for the transfer of 1 mol of amphiphilic block copolymer from solution to the micellar phase is given by the following equation, similar to that of non-ionic surfactant in the absence of electrostatic interactions [37]:

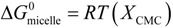
(1)
where *R* is the gas constant, *T* is the absolute temperature and *X*_CMC_ is the critical micelle concentration in mole fraction.

According to literature [37], the CMC was found at 3.2 × 10^−4^ g/mL for 4-arm star-shaped PEG–PCL block copolymers of similar molecular mass, namely 24,300 and 31,000 g/mol using the same indicator.

**Table 2:** CMC, molecular weight and standard free energy of micellization of PAAEAE copolymers.

Polymer	^a ^CMC (g/mL)	CMC (mol fr)	*M*_n_ (Da)	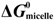 (kJ/mol)
DTE_DLA_mPEG5000	1.25 × 10^−4^	3.23 × 10^−9^	38,700	−41.3
DTE_DLA_PEG6000	1.25 × 10^−4^	3.53 × 10^−9^	35,400	−41.1
DTH_DLA_mPEG5000	2.50 × 10^−4^	1.17 × 10^−8^	21,400	−38.1
DTH_DLA_PEG6000	2.50 × 10^−4^	1.46 × 10^−8^	17,100	−37.6

^a ^Obtained from the variation curve of the absorbance of DPH at 356 nm as a function of polymer concentration in aqueous solution at 25 °C; *M*_n_ obtained from the GPC.

The free energies of micellization of PAAEAE copolymers are also shown in Table 2. The results presented negative values, which implied that the micellization of these copolymers in water was spontaneous. Moreover, with increasing the hydrophobic block length, the copolymer exhibited less negative free energies of micellization, also indicating a greater tendency to form micelles for PAAEAE copolymers containing DTE.

### 3.5. Dynamic Light Scattering (DLS)

**Figure 11:**
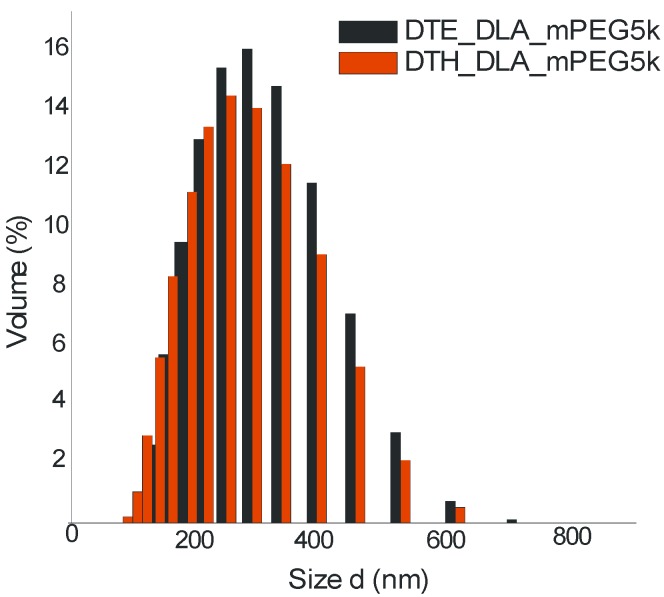
Mono-dispersed distribution for DTE_DLA_mPEG5000 (black) and DTH_DLA_mPEG5000 (red).

Particle size and polydispersity index (PDI) of nano-spheres formed from PAAEAE block copolymers were measured with dynamic light scattering (Figure 11). The nanosphere micelle sizes of PAAEAEs containing DTE and DTH were around 234 nm and 189 nm, PDI at 0.137 and 0.287, respectively. It was found that nanospheres had larger sizes for the copolymer containing DTE. These differences are probably related to the less hydrophobic nature and less branched structure of PAAEAEs containing DTE as compared to the longer hexyl moiety DTH. Importantly, both materials revealed mono-dispersed distribution of nanoparticles.

In comparison to earlier work of Kohn group [36], triblock copolymers containing mPEG5000, DTR (DTE and DTO—octyl ester derivative of tyrosine) and short-chain fatty acid, namely suberic acid (SA), showed nanosized structures ranging from 122 nm (DTE-SA/5000) to 55 nm (DTO-SA/5000) and PDI at 0.19 and 0.32, respectively.

### 3.6. TEM

**Figure 12:**
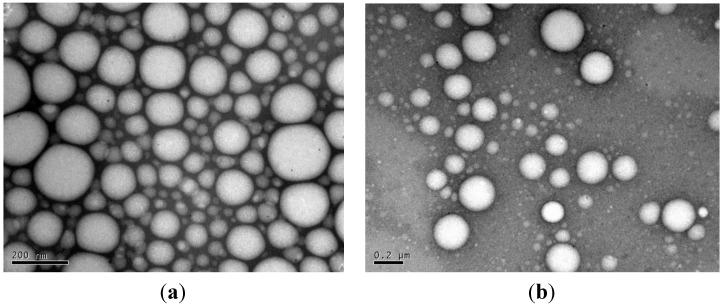
TEM images (negative staining) for (**a**) DTE_DLA_mPEG500 and (**b**) DTH_DLA_mPEG5000 nanospheres, respectively.

The morphology of nanospheres was determined using transmission electron microscopy (TEM). As can be seen in Figure 12, TEM pictures confirmed lower sizes and greater tendency of PAAEAEs to self-organize for the ethyl ester containing derivative of tyrosine as compared to hexyl ester.

## 4. Conclusions

We successfully synthesized new polymeric materials based on amino acids, fatty acids and PEG of different molecular masses. Spectroscopic methods revealed functional groups characteristic for poly(aliphatic/aromatic-ester-amide-ether). The GPC results demonstrated a random copolymer structure of the obtained materials as well as lower molecular masses for polymers containing the hexyl ester derivative of tyrosine and PEG of high molecular masses. We demonstrated self-organization of amphiphilic PAAEAE macromolecules in aqueous solution at CMC of 0.125–0.25 mg/mL, which revealed nanoscale spherical structures of 180–240 nm in diameter. The results obtained are promising for further work on self-organization of PAAEAE into well-defined micelles/nanospheres, which will be capable of encapsulating vitamins or antibacterial peptides, and finally applied as antibacterial coating on medical devices.
